# Sepsis caused by two *Phytobacter* species: clinical cases and polyphasic characterization of an emerging Enterobacteriaceae

**DOI:** 10.1128/asmcr.00051-25

**Published:** 2026-05-26

**Authors:** Marcelo Pillonetto, Maria Esther Graf, Geiziane A. Gonçalves, Debora N. O. Kulek, Ana Paula S. Chacon, Helki S. R. Pereira, Alexandre W. Losso, Mariana Bodnar, Polyana M. Miguel, Amanda Dal Lin, Letícia Kraft, Jose F. C. Neto, Geovana Vizentainer, Theo H. M. Smits, Fabio Rezzonico

**Affiliations:** 1 Paraná State Public Health Laboratory (LACEN-PR), São José dos Pinhais, Paraná, Brazil; 2 Pontifícia Universidade Católica do Paraná (PUCPR), Curitiba, Paraná, Brazil; 3 Hospital do Trabalhador - SESA- Secretaria de Saúde do Paraná, Curitiba, Paraná, Brazil; 4 Environmental Genomics and Systems Biology Research Group, Institute of Natural Resource Sciences (IUNR), Zurich University of Applied Sciences ZHAW, Wädenswil, Switzerland; Rush University Medical Center, Chicago, Illinois, USA

**Keywords:** polyphasic identification, *Phytobacter*, new species, emerging pathogen, sepsis

## Abstract

**Background:**

*Phytobacter* spp., a recently recognized human pathogen, presents significant diagnostic challenges. Conventional biochemical and automated identification methods often fail to distinguish *Phytobacter* from other *Enterobacterales*, which hinders accurate diagnosis and may potentially delay appropriate treatment. Since 2018, our state public health laboratory (LACEN-PR) has adopted a polyphasic identification strategy, integrating matrix-assisted laser desorption/ionization time-of-flight mass spectrometry, API 20E, targeted quantitative PCR, and whole-genome sequencing to investigate closely related *Enterobacterales* isolates that were initially misidentified and subsequently confirmed as *Phytobacter* spp. This study reports three cases of *Phytobacter*-associated sepsis, including a putative novel *Phytobacter* species and the first documented case of *bla*_NDM_-positive *Phytobacter ursingii*.

**Case Summary:**

Case 1: A 31-year-old man living with HIV developed sepsis following respiratory failure. Initial testing misidentified the isolate as *Leclercia adecarboxylata*, but the polyphasic approach revealed the potential for a novel *Phytobacter* species. The patient showed clinical improvement following targeted antimicrobial therapy. Case 2: A 50-year-old trauma patient developed sepsis caused by a *Phytobacter* spp. The isolate was closely related to that of Case 1. Despite treatment, he succumbed to the infection. Case 3: A 68-year-old woman with multiple comorbidities who developed septic shock. Blood cultures identified *bla*_NDM_-positive *Phytobacter ursingii*. The patient did not survive.

**Conclusion:**

These cases underscore the clinical significance of *Phytobacter* spp. as an emerging pathogen and highlight the challenges posed by their misidentification. The discovery of a novel species and multidrug-resistant *P. ursingii* highlights the need for improved diagnostic accuracy and effective treatment to enhance surveillance and improve patient outcomes.

## INTRODUCTION

Although *Phytobacter* spp. were isolated as early as the 1970s (1–3), they have only recently been recognized as human pathogens. Retrospective analyses suggest multiple strains previously assigned to other genera now fall within the *Phytobacter* clade (4–7). Some bloodstream isolates have been associated with high mortality (4, 8–10). Given the frequency of misidentification, it is likely that *Phytobacter* spp. have been involved in many unrecognized clinical infections and outbreaks worldwide.

*Phytobacter* spp. have been implicated in a range of infections, including sepsis, catheter-associated, pulmonary, and urinary tract infections (4, 11–13), as well as outbreaks in Brazil, the USA, Singapore, and Japan (5, 8, 11–13). Since then, leading authorities have emphasized the importance of accurate identification and surveillance of this genus (14–17).

Conventional identification methods, including biochemical tests (e.g., API 20E), automated systems (e.g., VITEK, Phoenix, and Microscan), and matrix-assisted laser desorption/ionization time-of-flight mass spectrometry (MALDI-TOF MS), often misclassify *Phytobacter* spp. as other *Enterobacterales* species (4–6, 11, 12). Due to inaccurate database entries, even advanced sequencing techniques can produce erroneous results, complicating diagnosis and species assignment (4, 7, 18). Due to phenotypic similarities and outdated databases, *Phytobacter* isolates are frequently misidentified as other members of the order Enterobacterales (such as *Pantoea agglomerans*, *Kluyvera intermedia*, *Leclercia adecarboxylata*, *Kosakonia* spp., *Cronobacter* spp., and *Citrobacter* spp.) (4, 6, 8, 13). These limitations compromise both clinical care and epidemiological investigations, emphasizing the need for polyphasic identification strategies and updated reference databases.

Here, we describe three cases of bloodstream infection caused by *Phytobacter* spp. in southern Brazil between 2020 and 2024. In addition to clinical summaries, we present a detailed microbiological investigation, including the discovery of a potentially novel species and the first report of *bla_NDM_*-positive *Phytobacter ursingii*.

## CASE PRESENTATION

Before presenting the clinical cases, we first summarize the Microbiological Characterization Workflow adopted in this study. Additionally, clinical cases are described and summarized in Table 1, which outlines the progression and key events of each case, and Table 2, which presents a comparative overview of microbiological and diagnostic test results.

**TABLE 1:** Chronological overview of clinical and laboratory findings in three *Phytobacter* sepsis cases^*b*^

Events	Case 1	Case 2	Case 3
Patient	31-year-old man	50-year-old man	68-year-old woman
Comorbidities	HIV infection	None reported	Hypertension, diabetes mellitus, and heart failure
Hospitalization^*a*^	March 2020	March 2020	January 2024
Antibiotic therapies^*a*^	Ceftriaxone 2 g IV q24h (day 0–7)	Cefazolin 2 g IV q8h (day 0–2)	Ceftriaxone 2 g IV q24h + Metronidazole 500 mg IV q8h (day 0–1)
	Azithromycin 500 mg PO q24h (day 0–5)		Piperacillin/Tazobactam 4.5 g IV q6h (day 0–3), then 2.25 g IV q8h (day 4–8)
	Oseltamivir 75 mg PO q12h (day 0–7)	Vancomycin 1.5 g IV q8h (day 0–1)	Micafungin 100 mg IV q24h (day 0–12)
	Sulfamethoxazole/Trimethoprim 1,600 mg/320 mg PO q6h (day 2–22), then 400 mg/80 mg PO q24h (day 23–25)		Acyclovir 400 mg PO q24h (day 0–9)
	Meropenem 1 g IV q8h (day 9–18)	Meropenem 2 g IV q8h (day 0–1)	Meropenem 1 g IV q8h (day 0–3), then 0.5 g IV q12h (day 4–5)
	Linezolid 600 mg IV q12h (day 9–11)		Linezolid 600 mg IV q12h (day 0–1)
	Polymyxin B 1,000,000 IU IV q12h (day 13–25)	Polymyxin B 1,000,000 IU IV q12h (day 0 only)	Polymyxin B 1,000,000 IU IV q12h (day 0–5)
	Fluconazole 200 mg IV q24h (day 18–20), then 150 mg IV q24h (day 21–28)		
Sample obtained^*a*^	Blood cultures (day 7)	Blood cultures (day 6)	Rectal swab collected during routine intensive care unit surveillance (day 4) and blood cultures (day 13)
Hospital lab results^*a*^	*Leclercia adecarboxylata* (day 9) AST (day 10)	*Leclercia adecarboxylata* (day 7) AST (day 7)	NDM-producing *Citrobacter freundii* for rectal swab (day 5) and carbapenem-resistant *“Pantoea agglomerans”* from blood culture (day 14)
LACEN-PR identification conclusion	*Phytobacter* sp.	*Phytobacter* sp.	*Phytobacter ursingii*
Clinical outcome^*a*^	Discharge (day 30)	Death (day 7)	Death (day 19)

^*a*^ The three hospitalizations occurred in the same institution.

^*b*^ “Day X” refers to the days of hospitalization. IV, intravenous; PO, per os; q24h, every 24 h; q12h, every 12 h; q8h, every 8 h.

**TABLE 2:** Identification workflow and results for *Phytobacter* isolates from the three clinical cases^*d*^

Test results		Case 1	Case 2	Case 3
LACEN-PR ID		27808 RM	27653 RM	50337 RM
Culture		MacConkey agar lactose positive	MacConkey agar lactose positive	MacConkey agar lactose positive
API 20E		1-2-0-4-7-3-3-7	1-2-0-4-7-3-3-7	1-2-0-4-5-3-3-7
		*Pantoea* spp. 2 (30.9%)	*Pantoea* spp. 2 (30.9%)	*Pantoea* spp. 2 (57.1%)
VITEK-2 AST-N panels (µg/mL)	Amoxicillin/clavulanic acid	ND	ND	ND
	Ampicillin	≥32 (R)	≥32 (R)	ND
	Ampicillin/sulbactam	≥32 (R)	≥32 (R)	ND
	Piperacillin/tazobactam	≤4 (S)	≤4 (S)	ND
	Ceftazidime	4 (I)	4 (I)	≥64 (R)
	Ceftriaxone	≥64 (R)	≥64 (R)	≥64 (R)
	Ceftazidime/avibactam	ND	ND	≥16 (R)
	Ceftolozane/tazobactam	ND	ND	16 (R)
	Cefepime	8 (R)	2 (I)	8 (R)
	Aztreonam	ND	ND	≤1 (S)
	Ertapenem	≤0.5 (S)	≤0.5 (S)	≥8 (R)
	Imipenem	≤0.25 (S)	≤0.25 (S)	ND
	Meropenem	≤0.25 (S)	≤0.25 (S)	≥16 (R)
	Amikacin	≤2 (S)	≤2 (S)	≥64 (R)
	Gentamicin	≤1 (S)	≤1 (S)	≥16 (R)
	Ciprofloxacin	≤0.25 (S)	≤0.25 (S)	≤0.06 (S)
Gradient concentration test^*a*^ (µg/mL)		Ceftazidime = 2 (I)	Ceftazidime R; Cefepime R;	ND
MALDI-TOF MS (VITEK-MS, IVD Myla database v4)		*Leclercia adecarboxylata* (99.9%)	*Leclercia adecarboxylata* (99.6%)	No identification
MALDI-TOF MS (VITEK-MS, RUO Saramis database v4.16; System v4.1.0.9, year 2010)		*Phytobacter* spp. (84.7%)	*Phytobacter* spp. (77.5%)	*Phytobacter* spp. (81.4%)
Full 16S rRNA gene^*b*^ - BLASTN and leBIBI (identical results)		*Pseudescherichia vulneris*	*Pseudescherichia vulneris*	*Phytobacter ursingii*
*bla*_CTX-M-2_ ^*b*^		Positive	Positive	Not detected
*bla*_NDM_ gene (in-house qPCR)^*c*^		Negative	Negative	Positive
*nif*L gene (in-house qPCR)		Positive	Positive	Positive
WGS and Type Strain Genome Server		*Phytobacter* sp. nov.	*Phytobacter* sp. nov.	*Phytobacter ursingii*

^*a*^ *E*-test (bioMérieux). This method was used as a complementary approach when VITEK-2 results were inconclusive.

^*b*^ The *bla*_CTX-M-2_ gene and the 16S rRNA were analyzed based on whole-genome sequencing (WGS) results.

^*c*^ The *bla*_NDM-1_ gene was detected by quantitative PCR (qPCR) and confirmed by WGS.

^*d*^ R, resistant; S, susceptible; I, susceptible, increased exposure (when applicable); N/A, not available; ND, not determined. Interpretive breakpoints for antibiotics were based on the BrCAST 2020 (case 01 and 02) and 2024 (case 03) guidelines (https://brcast.org.br/).

In 2020, the Paraná State Central Laboratory (LACEN-PR) issued a technical note highlighting the emergence of *Phytobacter* spp. and offering extended molecular identification and resistance screening for voluntarily submitted clinical isolates. A polyphasic identification strategy was adopted to clarify cases initially reported as *Leclercia*, *Pantoea*, or other rare taxa.

Isolates are typically received after the patient’s clinical course has ended; thus, LACEN-PR’s analyses are intended for epidemiological and scientific purposes rather than clinical decisions.

The identification workflow combined phenotypic and molecular methods. Initial screening was performed using MALDI-TOF MS (VITEK-MS) with both IVD (Myla software) and RUO (Saramis) databases, according to Mazzetti et al. (19), and API 20E (bioMérieux) for biochemical profiling. While *Phytobacter* is not included in the API 20E database, the system reliably excludes common *Enterobacterales* and remains a useful, low-cost preliminary tool. Alternate databases (https://bacdive.dsmz.de/api-test-finder; https://enteroplus.app.achillescdss.com.br) have recently included *Phytobacter* spp. phenotypes. A detailed characterization of *Phytobacter*’s biochemical traits, as determined using API 20E, is available in Smits et al. (4).

A *Phytobacter* genus-specific quantitative PCR (qPCR) assay targeting the *nif*L gene was previously proposed and validated. Briefly, this gene, which is part of the nitrogen fixation operon, was selected through comparative genomic analysis as a conserved and specific marker for the genus, due to its consistent presence in *Phytobacter* spp. and absence in closely related *Enterobacterales* (20).

Molecular confirmation involved genus-specific qPCR targeting the *nif*L gene, followed by 16S rRNA gene sequencing (MicroSEQ kit) and phylogenomic placement via whole-genome sequencing (WGS) (Illumina MiSeq), analyzed through TYGS (Type Strain Genome Server; https://tygs.dsmz.de/). The full-length 16S rRNA gene sequence was confirmed by WGS. Antimicrobial resistance genes, including *bla*_CTX-M_ and *bla*_NDM_, were identified via *in silico* analysis of WGS data. Antimicrobial susceptibility testing was performed using the VITEK-2 system (AST-N panel; bioMérieux) and interpreted according to the 2022 guidelines of the Brazilian Committee on Antimicrobial Susceptibility Testing (BrCAST).

This retrospective study focused on the microbiological characterization of emerging *Phytobacter* species. No patient intervention or prospective data collection was involved, and formal ethical approval was not required under local regulations. All data were fully anonymized, and only essential clinical and microbiological details are reported, without risk of patient identification.

### Case 1

A 31-year-old man with newly diagnosed HIV (CD4+ = 9  cells/µL; reference: 540-1731 cells/µL (21); HIV viral load = 149,858 copies/mL) was admitted with acute respiratory failure in March 2020. Empirical therapy for community-acquired pneumonia was initiated with ceftriaxone and azithromycin (day 1), followed by trimethoprim-sulfamethoxazole (day 6) for suspected pneumocystosis. On day 7, hemodynamic instability prompted the collection of peripheral blood cultures, which yielded a gram-negative bacillus.

On day 9, the hospital laboratory reported that the isolate was identified as *Leclercia adecarboxylata* by MALDI-TOF MS (VITEK-MS, using IVD database). Given ongoing clinical severity, therapy was escalated on day 10 to meropenem + linezolid, and polymyxin B was added on day 14 (for complete posology see Table 1).

Reanalysis using the RUO database yielded an 84.7% probability of *Phytobacter* spp. The API 20E biochemical profile was compatible with previously described *Phytobacter* phenotypes. A genus-specific qPCR targeting the *nif*L gene tested positive.

The full 16S rRNA gene sequencing identified *Pseudescherichia vulneris* as the closest match in both BLASTN and leBIBI analyses. WGS, analyzed via TYGS, confirmed it as a *Phytobacter* genus but did not correspond to any described species, supporting its classification as a putative novel species. WGS also revealed the presence of *bla*_CTX-M-2_, correlating with phenotypic resistance to cefazolin, cefuroxime, ceftriaxone, and cefepime. The isolate remained susceptible to ceftazidime, carbapenems, amikacin, and polymyxin. The patient improved and was discharged after 30 days.

### Case 2

A 50-year-old man was admitted to the intensive care unit (ICU) in March 2020 after polytrauma from a high-impact accident, including brain injury requiring decompression. On day 6, with signs of sepsis, empirical meropenem and vancomycin were initiated (for complete posology see Table 1), and blood culture was collected via peripheral venipuncture. Despite initial treatment, he died 7 days after admission.

Blood cultures yielded a gram-negative bacillus initially identified as *Leclercia adecarboxylata* by MALDI-TOF MS (VITEK-MS). Further analysis using the RUO database indicated *Phytobacter* spp. The API 20E biochemical profile was consistent with that of *Phytobacter*, and the *nif*L-targeted qPCR tested positive.

The full 16S rRNA sequencing provided inconclusive species-level resolution; however, WGS confirmed the isolate as a *Phytobacter* species closely related to that from Case 1 and also identified the *bla*_CTX-M-2_ gene, in agreement with phenotypic resistance to all tested cephalosporins, except ceftazidime. The isolate remained susceptible to carbapenems, amikacin, and polymyxin.

### Case 3

A 68-year-old woman with a history of hypertension, diabetes mellitus, and heart failure was admitted to the ICU in January 2024 with septic shock of presumed abdominal origin, associated with acute kidney injury requiring dialysis. Empirical ceftriaxone and metronidazole were initiated on day 1. Despite supportive care, her condition remained critical, leading to escalation to meropenem, polymyxin B, and linezolid. On day 5, a rectal swab (collected during routine ICU surveillance) tested positive for *Citrobacter freundii* harboring *bla*_NDM_.

On day 13, due to persistent critical illness, peripheral blood cultures (collected via peripheral venipuncture) were obtained.

The blood cultures yielded a carbapenem-resistant gram-negative bacillus, initially identified as *Pantoea agglomerans* by VITEK-2 (GN panel), while MALDI-TOF MS using the IVD database returned a “no identification” result. Analysis with the RUO gave an 81.4% chance of *Phytobacter* spp. The API 20E biochemical profile aligned with previously described *Phytobacter* patterns, and qPCR targeting the *nif*L and *bla*_NDM_ genes was positive. The 16S rRNA and WGS confirmed *Phytobacter ursingii* harboring *bla*_NDM-1_. Despite treatment, the patient died on hospitalization day 19.

## DISCUSSION

We report three cases of sepsis caused by two different *Phytobacter* species, including two attributed to a newly identified species pending validation and, to our knowledge, the first documented case of *bla*_NDM_-positive *P. ursingii*. These findings highlight the pathogenic potential of this emerging genus.

The two novel species isolates, recovered from ICU patients within 25 days, were closely related by WGS, suggesting nosocomial transmission (M. Pillonetto, L. N. V. S. Arend, D. O. Kulek, A. Mazzetti, S. Jordan, L. Fenske, J. Blom, M. T. Mira, T. H. M. Smits, F. Rezzonico, unpublished data). Their detection highlights the need for precise identification to support the recognition of outbreaks and public health responses.

In the third case, a *P. ursingii* bloodstream isolate carrying *bla*_NDM-1_ was detected. To our knowledge, carbapenemase production mediated by *bla*_NDM_ has never been reported in this species. *Phytobacter* spp. isolates are known to harbor a range of beta-lactamase genes, including *bla*_CTX-M_, *bla*_KPC_, *bla*_IMP-1_, *bla*_IMP-4_, *bla*_IMP-6_, *bla*_TEM_, *bla*_GES-5_, and *bla*_NDM_, further complicating antimicrobial treatment (4–7, 12, 22, 23) and reinforcing the importance of routine susceptibility testing.

**Fig 1:**
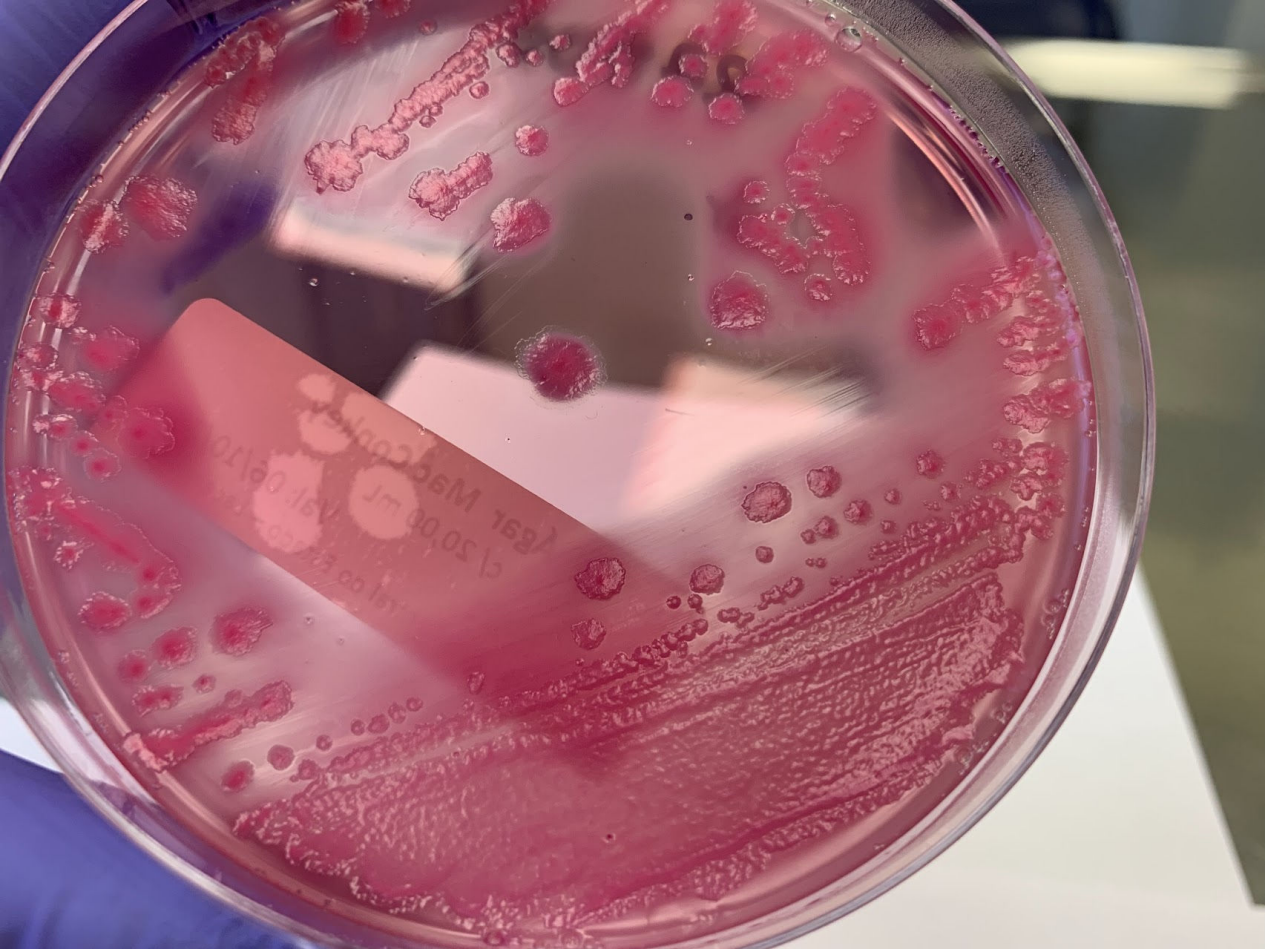
Growth of *Phytobacter* sp*.* nov. on MacConkey agar. Representative culture of *Phytobacter* sp*.* nov. showing strong lactose-positive colonies, surrounded by bile-precipitation on MacConkey agar after 24 h of incubation at 35°C, consistent with vigorous lactose fermentation. Colonies appear large, dry, and pink colored, with irregular margins. Morphology resembles *Escherichia coli* and/or *Citrobacter freundii* on MacConkey agar.

**Fig 2:**
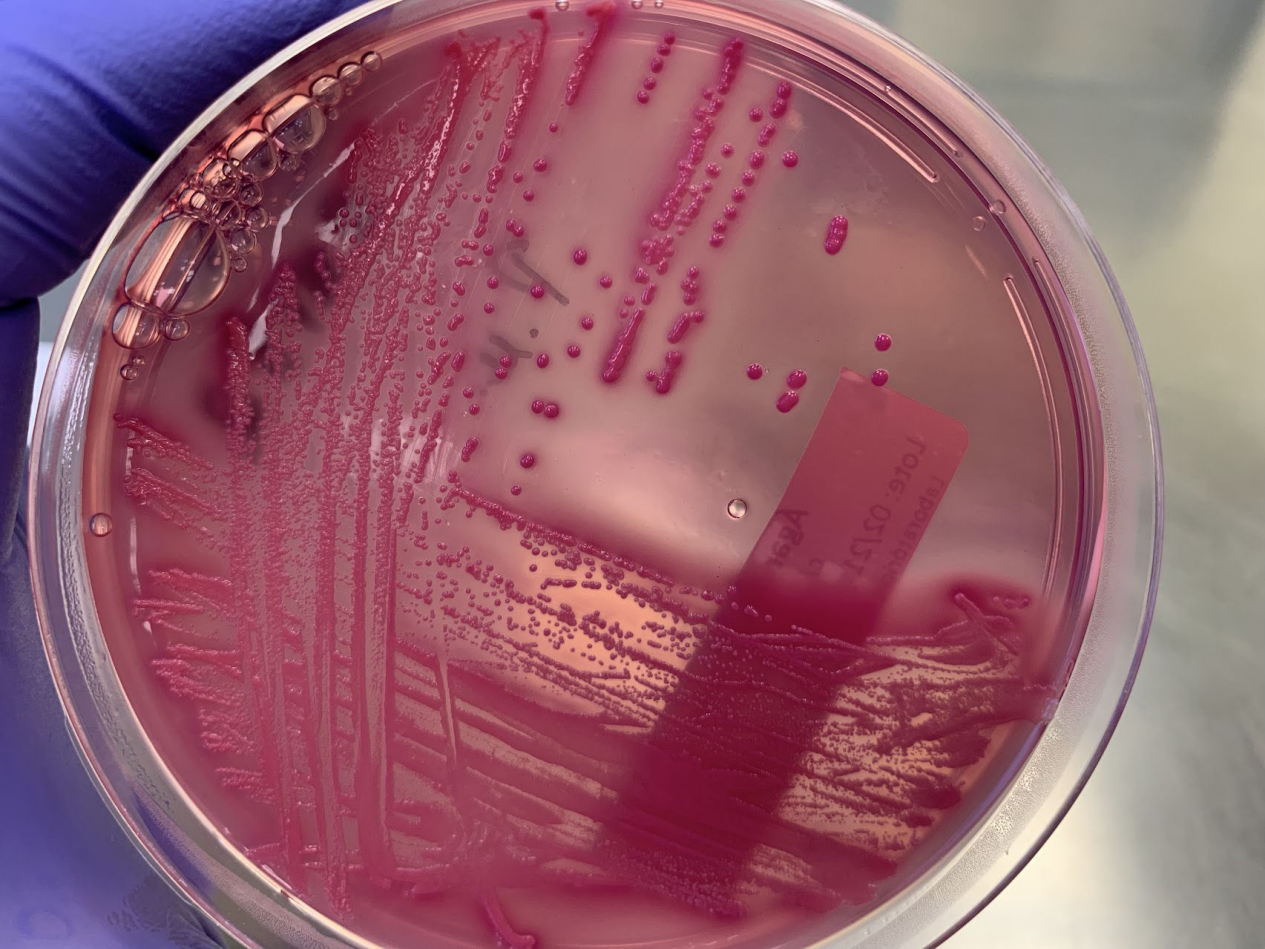
Growth of *Phytobacter ursingii* on MacConkey Agar. Representative culture of *Phytobacter ursingii* showing lactose-positive colonies on MacConkey agar after 24 h of incubation at 35°C. Colonies appear slightly mucoid, with regular margins and pink-colored, surrounded by bile-precipitation, consistent with strong lactose fermentation.

Clinicians and microbiologists should be aware of the diagnostic challenges associated with *Phytobacter* spp. Its colonies on MacConkey agar strongly resemble other common species such as *E. coli* and *C. freundii* (Fig. 1) or even *K. pneumoniae* (Fig. 2). Conventional methods, including MALDI-TOF MS, may fail to correctly identify isolates due to incomplete or outdated database entries for this genus, leading to misidentification as other *Enterobacterales* (24). Although RUO MALDI-TOF MS scores >80% may suggest *Phytobacter*, this alone is not sufficiently reliable. As described by Dubois et al. (25), scores between 60% and 99.8% qualify as “good ID” and may support presumptive identification; however, they are insufficient for accurate genus-level classification in clinical or epidemiological contexts. Until validated, *Phytobacter* spectra are incorporated into clinical databases; confirmation with genus-specific qPCR or WGS remains necessary.

Similarly, biochemical tests, such as API 20E, may also yield inconclusive or ambiguous results. Therefore, a polyphasic approach, combining phenotypic and genotypic methods, is essential for accurate identification. Specifically, the 16S rRNA gene sequencing, targeted qPCR assays for the genus *Phytobacter* (20), and WGS should be considered, especially in cases of severe infections or suspected outbreaks. WGS also enables the detection of resistance genes and virulence factors.

### Conclusion

In this case series, two of the three patients died during hospitalization. However, given their underlying conditions, critical clinical status, and comorbidities, it is not possible to directly attribute these deaths to a *Phytobacter* sepsis. Misidentification likely obscures the true burden of these infections. The genetic proximity of two ICU isolates supports *Phytobacter* as a potential opportunistic nosocomial pathogen, underscoring the need for accurate diagnostics and increased clinical awareness.

## Data Availability

The associated BioSamples and raw sequencing reads are publicly available under BioProject PRJNA1336074.
